# Scholarly publishing’s hidden diversity: How exclusive databases sustain the oligopoly of academic publishers

**DOI:** 10.1371/journal.pone.0327015

**Published:** 2025-06-26

**Authors:** Simon van Bellen, Juan Pablo Alperin, Vincent Larivière

**Affiliations:** 1 Chaire UNESCO sur la Science Ouverte, École de Bibliothéconomie et des Sciences de l’information, Université de Montréal, Montréal, Quebec, Canada; 2 Consortium Érudit, Montréal, Quebec, Canada; 3 School of Publishing, Simon Fraser University, British Columbia, Canada; 4 Public Knowledge Project, Simon Fraser University, British Columbia, Canada; 5 Observatoire des Sciences et des Technologies, Centre interuniversitaire de recherche sur la science et la technologie, Université du Québec à Montréal, Montréal, Quebec, Canada; 6 Department of Science and Innovation-National Research Foundation Centre of Excellence in Scientometrics and Science, Technology and Innovation Policy, Stellenbosch University, Stellenbosch, Western Cape, South Africa; Ingenio CSIC-UPV, SPAIN

## Abstract

Global scholarly publishing has been dominated by a small number of publishers for several decades. This paper revisits the data on corporate control of scholarly publishing by analyzing the relative shares of scholarly journals and articles published by the major publishers and the “long tail” of smaller, independent publishers, using Dimensions and Web of Science (WoS). The reduction of expenses for printing and distribution and the availability of open-source journal management tools may have contributed to the emergence of small publishers, while recently developed inclusive databases may allow for the study of these. Dimensions’ inclusive indexing revealed the number of scholarly journals and articles published by smaller publishers has been growing rapidly, especially since the onset of large-scale online publishing around 2000, resulting in a higher share of articles from smaller publishers. In parallel, WoS shows increasing concentration within a few corporate publishers. For the 1980–2021 period, we retrieved 32% more articles from Dimensions compared to the more selective WoS. Dimensions’ data showed the expansion of small publishers was most pronounced in the Social Sciences and the Arts and Humanities, but a similar trend is observed in the Natural Sciences and Engineering, and the Health Sciences. A major geographical divergence is also revealed, with English-speaking countries and/or those located in northwestern Europe relying heavily on major publishers for the dissemination of their research, while the rest of the world being relatively independent of the oligopoly. Finally, independent journals publish more often in open access in general, and in Diamond open access in particular. We conclude that enhanced indexing and visibility of recently created, independent journals may favour their growth and stimulate global scholarly bibliodiversity.

## Introduction

Global scholarly publishing has been dominated by a restricted number of publishers since at least the 1970s [1]. The market share of the world’s largest publishers, which include Elsevier (part of RELX), Springer Nature, Wiley, Taylor & Francis, and Sage Publications, in the global production of scholarly journals and articles has grown continually since the 1970s [2]. Using the Web of Science (WoS), Larivière *et al*. (2015) quantified the growing share of publications controlled by those publishers, in terms of numbers of scholarly journals and articles, the latter attaining more than 50% in 2013 [1]. They also showed how those proportions varied across disciplines, with the Social Sciences being the most highly concentrated (70%) and the Humanities remaining relatively independent (20%). Most papers in Medical and Natural Sciences, where journals are the most expensive [3] and publication volumes are highest, were controlled by the five major publishers.

Since the Larivière *et al*. paper published a decade ago [1], the oligopolistic structure of the scholarly journal ecosystem has been used as a reference framework in many studies [4–8]. Since the start of the digital era, scholarly publishing has evolved rapidly, characterized by changes in dissemination patterns and the rise of open access (OA) [9]. The last decades have also seen significant progress in open knowledge practices and applications, driven by both technology and a sociocultural willingness of scholars to render new knowledge more accessible [10]. While the dominance of the oligopoly and its important repercussions on the scholarly publishing ecosystem have become “common knowledge”, it remains critical to track how the global share of the major publishers evolves. We thus aim to re-evaluate the level of concentration of scholarly journals and articles, using tools that were not available or nascent only a decade ago, but which have proven to provide an alternative to WoS and Scopus for bibliometrics.

Founded in 2004, Google Scholar has been explored as a source for bibliometric analysis, likely favoured by its accessibility and its high performance in detecting content. Nevertheless, Google Scholar’s strength also represents its drawback: including a wide range of sources, it generally lacks the quality control needed for robust bibliometric analyses [11]. Launched in 2018 and 2022, respectively, Dimensions [12,13] and OpenAlex [14] rely on increasingly powerful algorithms, the growth in use of persistent identifiers and the openness of article metadata, mostly through Crossref. Dimensions and OpenAlex follow a similar approach to indexing and have similar coverage [15–17]. These databases offer an opportunity to re-assess the extent to which major publishers control the research dissemination landscape without the limitations of incomplete coverage of the more selective bibliographic databases, such as WoS and Scopus [18]. Applying selective criteria for inclusion, WoS and Scopus purposely index a restricted set of journals which represents a subset of all scholarly journals published worldwide, as suggested by comparisons with Dimensions [19] or Ulrichsweb [20]. The criteria for inclusion in WoS and Scopus are more generally met by journals published by commercial publishers and may be difficult to fulfill by smaller, independent publishers, as they are based on technical and editorial characteristics, a preference for the use of English, and their level of “international impact”, quantified by citations. WoS’ and Scopus’ collections are biased towards the Natural and Health Sciences, English-language content [20,21] and journals and publishers from North America and Europe. Recent studies have quantified the major commercial publishers’ market share. Eger and Scheufen [22] showed a slight decline in the “traditional” five major publishers’ share of journals between 2006 and 2019, which was attributed to a decline in Elsevier’s journal portfolio. Shu and Larivière [4] used restrictive (WoS) and inclusive (Dimensions) databases to assess the level of concentration in the OA publishing market. They showed how that market is increasingly concentrated in WoS – which suggests concentration in the elite set of journals – while it is decreasing for most of the period in Dimensions.

The aim of this article is to map the current structure of scholarly publishing in terms of corporate control, its disciplinary and geographic distribution, and the adoption of OA, thus providing an update on the state of the oligopoly of commercial publishers. Using the inclusive Dimensions database and the rather exclusive WoS, we also aimed to compare indexing levels of the major publishers’ journals and articles with those of the smaller publishers. In addition, we documented two article characteristics, author origins and OA status, as a function of the publisher status.

## Background

Although commercial practices have been common in scholarly publishing since the early 19th century [23], the dominance of the major publishing firms became obvious during the 1990s [1], leading to what is commonly referred to as the “serials crisis” [24]. Coinciding with the rise of the major commercial publishers, the large-scale development of digital, online publishing may well be considered the most significant economic shift in scholarly publishing over the past century. Digital publishing appeared beneficial to major commercial publishers as it allowed them to increase profit margins. As detailed by Larivière *et al*. [1], the abolishment of print and the potential for Web downloads have enabled publishers to sell unlimited additional copies to a growing readership without incurring extra costs. The effects of digital publishing may have been reinforced as content became increasingly available in OA after 2000 [9] and therefore accessible to a much greater readership. Yet, for users, this potential growth in accessibility came at a cost. Zhang *et al*. [25] have shown that commercial publishers’ revenues have been increasingly relying on Article Processing Charges (APC) rather than subscriptions; this business model has been the driving factor for recent mergers and acquisitions observed in the industry. This is exemplified by the recent acquisition (2021) of Hindawi by Wiley, or by the investments made by the Holtzbrinck Publishing Group, now the major shareholder of Springer Nature, in Frontiers media [4]. Drawing on journal’s APC price lists for the five most prolific publishers, in terms of papers (Elsevier, Springer Nature, Wiley, Taylor & Francis and Sage Publications), Butler *et al*. [6] quantified the revenue stream coming from APC, which exceeded more than 1 billion $US for 2015–2018. In recent years, relatively young, APC-only publishers, such as MDPI and Frontiers, have caught up with the traditional major publishers in terms of article volumes [8]. Haustein *et al*. [26] estimate that the APC revenue of these publishers, along with Elsevier, Springer Nature, and Wiley, approached 2.5 billion $US in 2023. Digital OA publishing also contributed to the rise of questionable (so-called predatory) journals, i.e., those that do not adhere to ethical publication practices [27,28].

However, online publishing also favoured the creation of new, independent journals, as the reduction of the expenses related to distribution made production and dissemination of scholarly journals more cost-effective for newly established journals. Here, we refer to journals that share multiple characteristics with those described by Bosman *et al*. [29] in a recent study on Diamond OA, i.e., OA journals that do not require APCs: scholar-managed, nationally focused, running on small budgets and publishing relatively low volumes of articles, often in languages other than English. Founding such journals was likely facilitated by the development of open publishing software, such as Open Journal Systems (OJS; launched in 2002), Janeway, Fulcrum and Scholastica [30], which allowed editorial teams to streamline manuscript handling and enhance discoverability and indexing of published content.

The digital era also saw the development of national publishing platforms. Often aligned with evolving national research policies and motivated by a willingness to enhance the social impact of research, several countries have created platforms for national, independent journals, acknowledging the importance of publishing in national languages and, typically, in OA [31,32]. Naturally, such infrastructures have contributed to the long-term viability of independent journals. These platforms, including Redalyc (originally from Mexico), SciELO (originally from Brazil), OpenEdition (France), Érudit (Canada), Journal.fi (Finland) and Hrčak Portal (Croatia), among others, strongly vary in age, size and geographic coverage. While SciELO and Redalyc together represent around 800,000 scholarly OA articles from Latin America [33], journal.fi provides access to around 50,000 articles from Finnish journals [31].

Tracking these publications is complicated by their rare presence in the main bibliometric databases and indexes, such as WoS and Scopus [34]. Focusing on Diamond OA journals, Bosman *et al*. [29] stressed that “[t]he most challenging area for OA diamond journals is indexation and content visibility in the main international indexes”. In 2021, around 25,000 scholarly journals used OJS, mostly located in Asia and the Pacific, and Latin America and the Caribbean states and a public dataset suggests more than 40,000 journals actively used OJS in 2024 [35]. Based on the various initiatives to support independent journals that exist globally, we expect the total number of independent scholarly journals to be considerable.

## Materials and methods

As a first step, data was retrieved from Dimensions and WoS databases to compare the collection sizes, in terms of articles and journals. Dimensions was accessed using Google BigQuery and the September 1, 2024 snapshot was used for analyses. We excluded documents that were not marked as type = ‘article’ which eliminated books, book chapters, proceedings, monographs and preprints. We further filtered “non citable” content, such as editorials, book reviews, comments, etc. Access to WoS was obtained through the Observatoire des sciences et des technologies at Université du Québec à Montréal. Data was extracted from its three main indexes, the Science Citation Index Expanded, the Social Sciences Citation Index and the Arts & Humanities Citation Index. Like the Dimensions dataset, the WoS dataset was limited to original articles and review articles. For both databases, we only included journals having an ISSN. Initial analyses based on a corpus of documents having a DOI were not conclusive, because WoS appeared to have very low numbers of indexed articles identified by a DOI before the digital era. The period of analysis was set initially at 1980–2023; however, it was later restricted to 1980–2021, as markedly lower numbers of documents were retrieved for both 2022 and 2023, suggesting a delay in indexing, possibly creating a biased dataset for these years.

Information on journals’ publishers — and especially on their ownership structures — often remains ambiguous or incomplete in bibliometric databases. Therefore, we performed data cleaning and reorganization, mainly to take account of the presence of varying forms referring to the same publisher. For example, WoS was found to use both “Wiley” and “Wiley-Blackwell” for the same publication year (S1 Table). Likewise, at total of 73 variants were found for Elsevier, which, once standardized, totalled close to 10 million articles. As we aimed to quantify the proportions of articles and journals published by the major publishers, relative to the content of other publishers, data cleaning efforts specifically considered the group of major publishers. We also took, to some extent, account of mergers and acquisitions, the most notable being Lippincott Williams & Wilkins, which was integrated in Wolters Kluwer, and Plenum Publishing Corporation, which was merged with Springer Nature. Merging implied at most a few hundreds of thousands of articles; therefore, these mergers had very small effects on total numbers and general trends. Some of the content in Dimensions was associated with platforms and aggregators, such as JSTOR, SciELO and CAIRN, rather than publishers. As we focused on publishers’ concentrations, these entities were not included in the rankings of the degree of concentration, but their articles were accounted for in calculating the publishers’ degree of concentration. The content of some questionable publishers, such as OMICS, Sophia Publishing Group and Academic Journals, was removed from the datasets, based on an updated version of Beall’s list (S1 Table). However, some questionable content is likely to have been included as the evaluation could not be performed in high detail. Moreover, it is increasingly recognized that various questionable practices exist and that they form a continuum [36]; it is therefore unrealistic to class individual journals dichotomously in this respect, and highly complex to justify their exclusion from the dataset.

As the publishing landscapes of the Natural Sciences and Engineering, the Health Sciences, the Social Sciences, and the Arts and Humanities exhibit important differences in terms of corporate control [1], dissemination languages [37] and OA [9], among others, we presented results specifically for each of these major disciplines. Field classifications are specific to each database, with Dimensions using automatic classification of articles through algorithms while WoS relies on journals. In Dimensions, articles may be assigned to multiple fields of research. As the analyses of discipline-specific trends implied only relative data (i.e., quantification of relative shares of articles between major and smaller publishers), we did not attempt to re-classify articles to a single field. OA prevalence was quantified based on two sources. At the article level, data from Unpaywall [38], integrated in Dimensions, was used to identify the access status, including “Gold”, “Hybrid”, “Bronze” and “Green” OA, as well as “Closed” content, following the definitions by Piwowar *et al*. [9]. Articles marked as Gold OA include both articles published with and without the payment of APC. As noted earlier, we refer to the latter as “Diamond” OA, while the former may be considered “real” Gold. In addition, we used the data provided by DOAJ to map the adoption of OA at the journal level. Indexing the use of APC, DOAJ allows for the distinction between Gold and Diamond OA journals. It should be noted, however, that open publications, either Gold or Diamond, may be classified as Bronze OA if a Creative Commons licence is not detected by Unpaywall.

## Results

### The divergence of bibliometric databases

**Fig 1:**
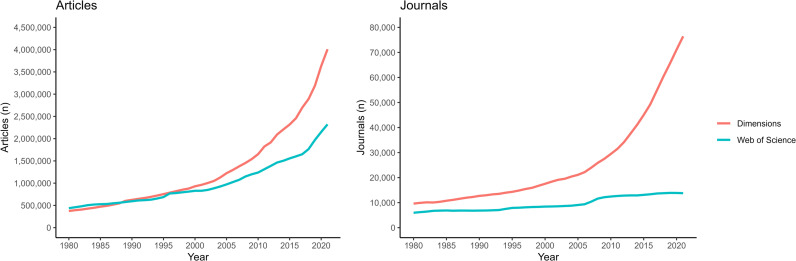
Trends in the numbers of articles and journal titles published annually, according to Dimensions and WoS, 1980-2021.

The number of scholarly articles published between 1980 and 2021 is 32% higher according to Dimensions compared to WoS, with Dimensions containing around 55 million articles, while WoS returned close to 42 million articles. Differences in the numbers of journals are more distinctive, as Dimensions indexes a total of 94,248 journals that were active at one point between 1980 and 2021, compared to 19,445 journals with activity in WoS. While Dimensions shows an accelerating growth in journals since 2000, WoS suggests only a slight increase during the last 25 years (Fig 1). In terms of articles, both databases return increasing numbers since 1980, but growth appears much quicker when observed through Dimensions. This suggests that the growth of WoS has not followed that of scholarship [39] and that its indexing gap is growing. Overall, the divergence between Dimensions and WoS is much greater for journals than for articles, which implies that WoS has an indexing advantage for journals with high publishing volumes.

### The share of major publishers

**Fig 2:**
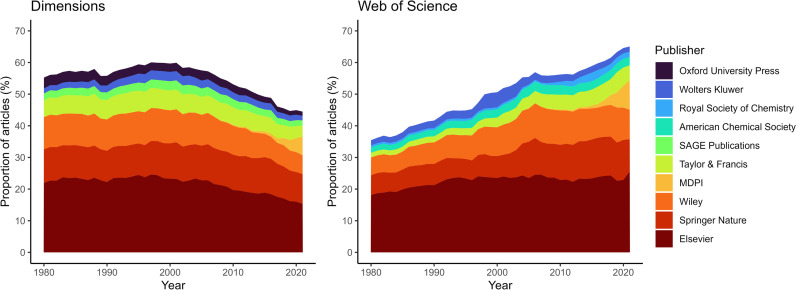
Proportion of articles published by a selection of major publishers, for those that have been part of the five major publishers at some point between 1980 and 2021, for each database.

The data from Dimensions reveal the important presence of the major publishers, but their share of all scholarship has been decreasing since the late 1990s (Fig 2). This represents a markedly different image compared with the data from WoS, which shows an ongoing, apparent increase in the concentration of scholarly journal articles among the major publishers. Dimensions and WoS converge regarding the relative stability of the *composition* of the group of major publishers: Elsevier, Springer Nature, Wiley, MDPI and Taylor & Francis are the current five largest publishers in terms of the volume of articles, and except for MDPI, they have been among the main publishers since at least the 1980s (Fig 2; Table 1). Both databases register the rapid growth of MDPI, a commercial OA publisher that emerged after 2010, which has become the fourth publisher in terms of article numbers.

**Table 1:** Numbers of articles for the major publishers, according to Dimensions and WoS.

Publisher	1980-1989		1990-1999		2000-2009		2010-2021	
	Dimensions	WoS	Dimensions	WoS	Dimensions	WoS	Dimensions	WoS
Elsevier	1,079,517	1,020,620	1,770,923	1,611,626	2,666,073	2,327,488	5,418,462	4,688,464
Springer Nature	481,197	329,535	765,821	456,881	1,334,754	941,346	3,211,434	2,357,551
Wiley	468,507	306,739	762,987	540,132	1,180,638	1,007,526	2,208,895	2,017,908
MDPI	0	0	229	21	7,893	4,548	686,090	602,745
Taylor & Francis	272,708	85,249	472,419	159,924	696,451	382,826	1,412,795	1,041,457
SAGE Publications	109,277	24,686	192,946	41,910	327,487	153,727	652,065	474,613

Specifically focusing on the share of the five major publishers, Dimensions shows a decreasing concentration of articles since the late 1990s, declining from 65% in 1989 to 44% in 2021. In WoS, this share went up from 33% in 1980 to 59% in 2021. Trends are similar for the top 20 publishers. Only exception to this trend, WoS shows a slight decrease in the share of the major publishers (Fig 2), reflecting an increase in the number of journals indexed, around 2007–2008 (Fig 1). This interruption was caused by a one-off addition of journals, relatively frequently associated with the Social Sciences and the Arts and Humanities. During recent years (2019), WoS suggests the four major publishers account for more than half of the papers published. According to Dimensions, it would take 11 publishers to attain the same proportion of articles.

**Fig 3:**
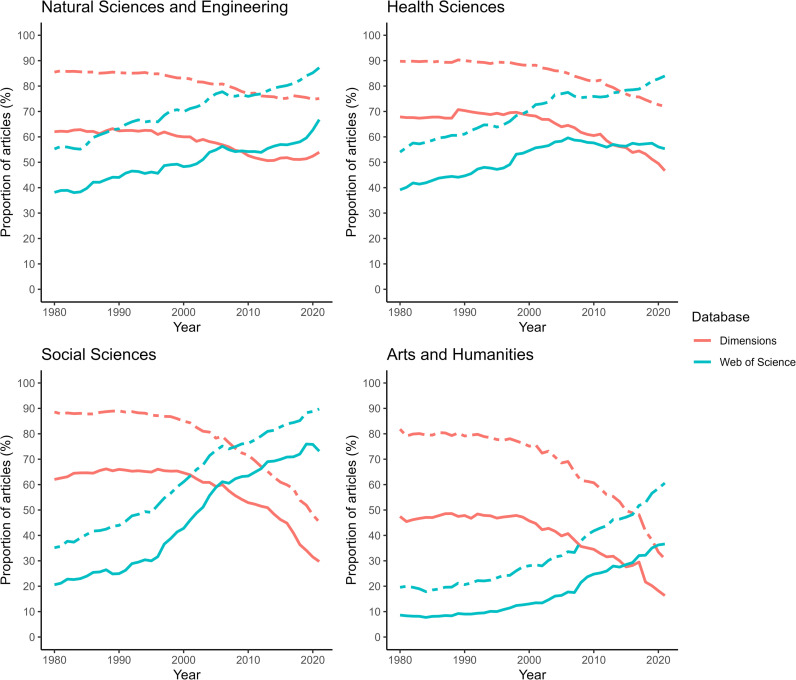
Proportions of articles published by the main publishers, per major discipline, according to two bibliometric databases, 1980-2021. The solid line represents the share of the five major publishers, and the dashed line shows the share of the 20 major publishers, for each year.

Breaking up the data by discipline, the decreasing shares of the major publishers appear to be more pronounced in the Social Sciences and the Arts and Humanities in comparison with the Natural Sciences and Engineering, and the Health Sciences (Fig 3). It also appears Dimensions and WoS diverge more significantly for the Social Sciences and the Arts and Humanities. Dimensions does not only return higher *total* numbers of articles; it also returns higher numbers of articles for the five major publishers, showing that a certain number of journals published by the major publishers have not been indexed in WoS (Table 1). For the publication years 2019–2021, this was particularly the case in the Arts and Humanities, where WoS retrieved only 44% of the number of major publishers’ papers returned by Dimensions; these proportions were 72% in the Social Sciences, 77% in the Natural Sciences and Engineering and 97% in the Health Sciences.

The declining proportion of articles and journals published by major publishers, shown by Dimensions, reflects the growth in independent journals’ papers outpacing those of the major publishers, especially in the Arts and Humanities, and the Social Sciences. The growth in independent journals has become perceivable through the more inclusive indexing approach of Dimensions compared to WoS and can be associated with the advent of digital publishing. Out of a total of 82,126 active journals (2019–2021) present in Dimensions, 70,550 journals are not associated with a major journal publisher, confirming Dimensions effectively sheds light on journals independent of large publishing structures. As an example, among the 82,126 journals, we found, matching by ISSN, 28,246 (34%) journals that use OJS’ open-source software.

### Geographical divergences in the dominance of main publishers

**Fig 4:**
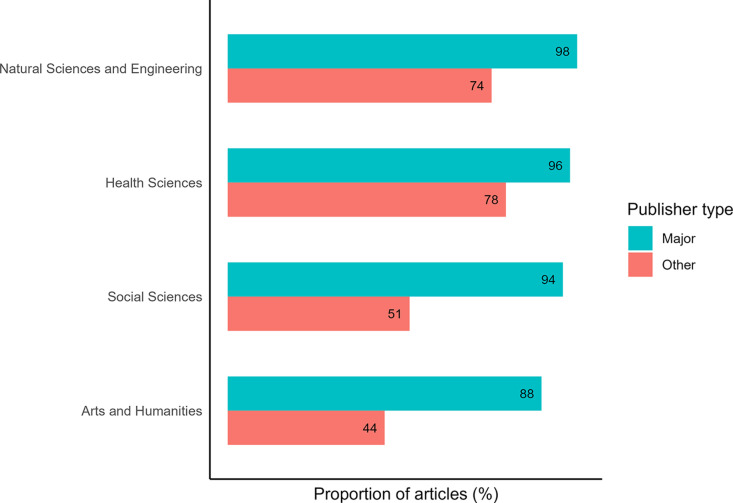
Proportion of articles published having a country of affiliation indexed for the first author in Dimensions, for the five major publishers (based on the number of articles, per discipline) and independent publishers, per discipline (2019-2021).

The retrieval of author affiliations from Dimensions revealed major divergences in indexing levels. For articles published by the five major commercial publishers, we found between 98% (Natural Sciences and Engineering) and 88% (Arts and Humanities) of the articles had a country of affiliation identified for their first author (Fig 4). Proportions were lower for independent publishers, and the difference with the major publishers appeared particularly important in the Social Sciences (51%) and the Arts and Humanities (44%).

**Fig 5:**
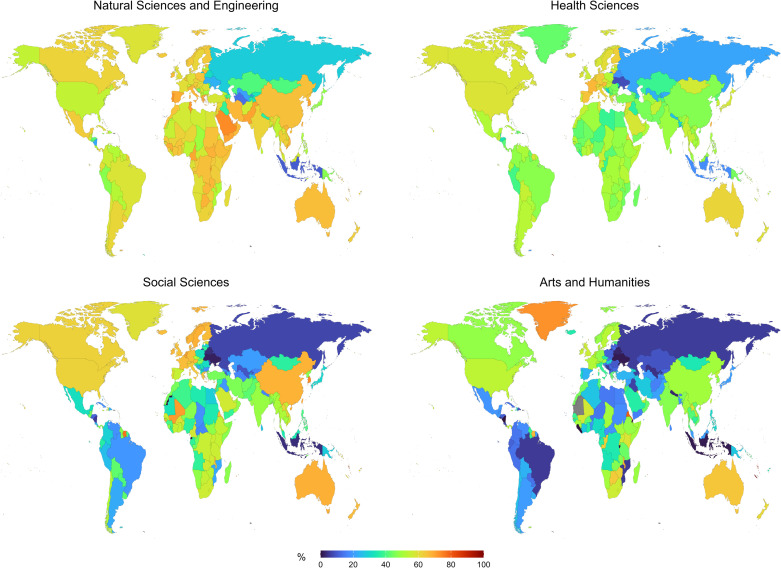
Proportion of articles published by the five major publishers (based on the number of articles, per discipline), based on data from Dimensions (2019-2021). Articles were attributed to a country according to the origin of the first author. Map layout from Natural Earth (public domain).

Despite incomplete affiliation data, it is evident that, in addition to the variations associated with disciplines and journal origins, there is a strong geographic divergence in the use of the major commercial publishers’ journals by researchers. In the Natural Sciences and Engineering 80% of the world’s countries publish at least half of their papers in journals hosted by the major publishers (Fig 5). Nevertheless, several countries show much lower proportions, particularly Indonesia (10%), Ukraine (24%), Russia (28%) and Japan (48%), along with many central Asian and central American countries. In the Health Sciences, overall reliance on major publishers is smaller, but also rather polarized. Whereas many countries publish high numbers of articles in the major commercial publishers’ journals, such as France (67%), Germany (63%), the Netherlands (62%) and the United States (59%), several countries in the Global South publish relatively frequently in smaller publishers’ journals, such as Bangladesh (30%), Libya (38%) and Niger (40%).

The relative use of commercial publishers’ journals by researchers is even more contrasted in the Social Sciences and the Arts and Humanities (Fig 5). In the Social Sciences, many countries show a very limited use of commercial publishers’ journals for the publication of their research, with 24% of the countries each having less than 30% of their articles published by these publishers. These countries are mostly located in Latin America, eastern Europe, central Asia and parts of northern Africa, in addition to Indonesia (4%) and Russia (7%). Still, western Europe, North America and China generally show values well exceeding 50%. This contrasting trend is similar in the Arts and Humanities, yet the commercial publishers’ share is still lower overall.

### Open access and publisher types

**Fig 6:**
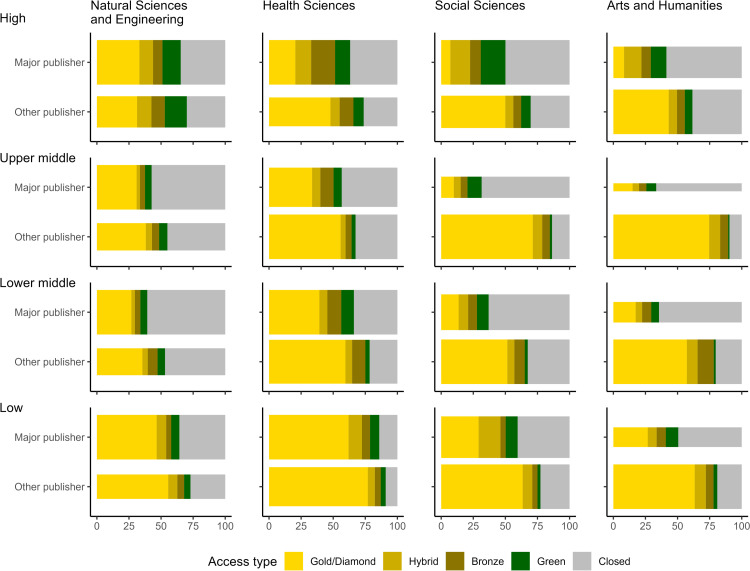
Authors’ use of different types of OA, quantified by the number of articles (%), as a function of their country of affiliation income class based on gross national income (GNI) per capita (high/upper middle/lower middle/low), the presence of major publishers and the discipline. Income classes were calculated using the World Bank Atlas method (2023), using data from https://datahelpdesk.worldbank.org/knowledgebase/articles/906519-world-bank-country-and-lending-groups, downloaded on November 5, 2024. Bar width represents, within each panel, the relative article sample size for the major/other publisher.

Combining Dimensions’ output and OA status information from Unpaywall, it appeared articles published by the smaller publishers are more frequently published (Gold/Diamond, Hybrid), or available (Bronze, Green) in OA, when taking account of the author’s country income class and discipline (Fig 6). Journals independent of the major publishers are particularly frequently published in Gold/Diamond OA, a category which includes both articles published with and without payment of APC (Fig 6). The smaller publishers’ OA advantage, compared to the major publishers, is more pronounced in the Arts and Humanities and the Social Sciences, with differences markedly smaller in the Health Sciences and rather subtle in the Natural Sciences and Engineering. We also found that OA prevalence decreases as GNI per capita increases, both for articles published at major publishers and for those published by smaller, independent publishers. This trend is disrupted by authors from upper middle-income countries, who publish particularly frequently in Gold/Diamond OA in independent journals of the Social Sciences and the Arts and Humanities, with Brazil, Argentina and Colombia standing out, each showing an OA prevalence exceeding 80%. These high proportions can likely be attributed to the presence of OA mandates and publishing platforms covering research activity in these countries and major parts of Latin America in general. These data show that independent publishers’ journals, besides being important venues for researchers from lower income countries, also lead more frequently to OA publications.

**Fig 7:**
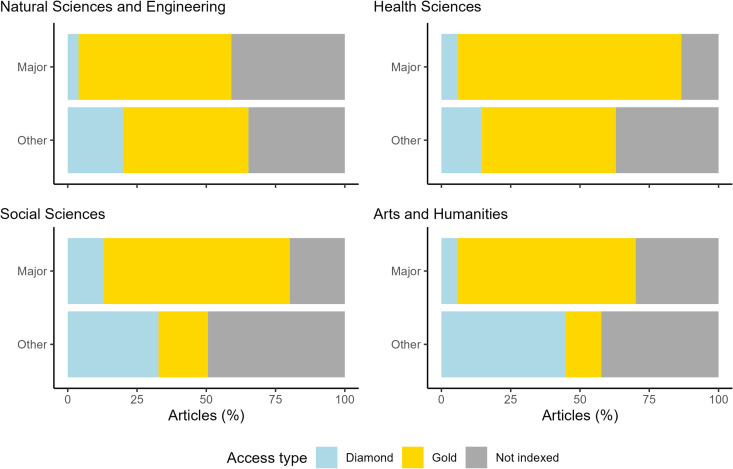
Proportion of article publishing modes and DOAJ indexing for major and other publishers’ OA journals, per discipline, 2019–2021.

We used DOAJ to specify the prevalence of APCs among OA journals. Combining DOAJ data with data extracted from Dimensions, it appears smaller publishers’ use of APCs is less common compared to the major publishers’ journals, particularly in the Arts and Humanities (Fig 7). Considering articles published in DOAJ-indexed journals, 77% of those published by smaller publishers in the Arts and Humanities are identified as Diamond OA, compared to 8% for the major publishers’ journals. Differences are smaller for the other disciplines, yet still significant: 65% vs. 16% in the Social Sciences, 23% vs. 7% in the Health Sciences and 31% vs. 7% in the Natural Sciences and Engineering. As potentially high numbers of openly accessible articles are published in open journals that are not indexed in DOAJ, we also quantified the proportion of Gold/Diamond OA articles (as defined by Unpaywall) published in journals not indexed in DOAJ. The proportion of open articles published in Gold/Diamond journals absent from DOAJ was 37%, and higher for the smaller publishers’ journals, in all disciplines, except for the Natural Sciences and Engineering. These results may reflect the difficulty for many of the smaller publishers’ journals to become registered in DOAJ.

## Discussion

### The current state of the oligopoly

The oligopoly of corporate publishers persists at a global level, but its dominance is partial and has been declining for decades. It appears strong according to the selective database WoS and it prevails in western Europe, North America and China, and especially in the Natural Sciences and Engineering and the Health Sciences. In parallel, however, the major publishers are much less dominant for many countries in the Global South, such as Brazil, Indonesia and Russia. These important differences between countries may have multiple causes. In some cases, countries have strongly centralized scientific policies and well-established support for domestic, mainly not-for-profit journals, which is the case in both Indonesia and Brazil. On the other hand, strong national incentives to publish in “high-impact” international journals may be as frequent, for example in China [40], which only recently abolished direct financial rewards for its researchers to this effect, but also in some western countries [41,42]. Countries having English as a national language, or having a strong English-speaking population, such as Australia, New Zealand, South Africa, Kenya, Tanzania and Ghana, may have a natural penchant for major commercial publishers’ journals, while a long history of pursuing research in national (non-English) languages may contribute to some countries refraining from publishing among commercial publishers. The latter may be the case for Russia and countries in Eastern Europe.

### Smaller publishers’ journals and perceptions of quality

In the context of digital publishing, we may consider questioning the relevance of the traditional dissemination and archiving functions of the journal as an entity [43], as, in the digital era, access to articles generally bypasses the journal’s cover page. Likewise, we may question the relevance of the journal’s *prestige* in estimating article “quality” or “impact”, in line with the core philosophy of the DORA and CoARA initiatives. The long tail of scholarly journals, represented by journals independent of commercial structures, typically indexed in Dimensions, but not in WoS, are often perceived as “less relevant” or even “low quality”, as they lack an Impact Factor, or, more generally, because of the lower numbers of citations these journals may receive. Two phenomena contribute to this perceived lower relevance or quality. First, citations reflect reuse by the scientific community, and it is not surprising the level of reuse is higher for the relatively well-indexed content of the Natural Sciences and Engineering; research in the Social Sciences and the Arts and Humanities is more nationally and locally focused compared to the Natural Sciences and Engineering [44,45], which explains their articles have a smaller readership that could potentially cite them. As a result, articles of these disciplines appear to have a lower “global impact”, yet they find their utility in addressing issues embedded in a geographically, socially or culturally restricted context. A second phenomenon explaining the lower perceived relevance of smaller publishers’ journals is related to the indexing prevalence itself: journals that are present in major databases are more easily retrieved, which should lead to a higher number of reads, and likely higher number of citations. Thus, indexing in exclusive databases leads to a perceived higher relevance or quality of journals, which further enhances the prestige attributed to the journal and its content; it therefore acts as a strong positive feedback mechanism.

### Journal indexing

Despite the potential of online publishing, the visibility of journals not linked to major publishers remains an issue in the digital era. Indexing bias is not exclusive to WoS; we found DOAJ presents a more complete picture of major publishers’ journals compared to smaller publishers, corroborating earlier findings by Bosman *et al*. [29]. Likewise, the highest-quality distinction within DOAJ, the now defunct DOAJ Seal, was most often attained by commercial publishers [8]. Indexing criteria, whether applied by WoS, Dimensions, DOAJ, Crossref or other databases and service providers, often comprise a range of practices in journal management and the adoption of machine-readable metadata including the use of persistent identifiers. These are requirements many independent journals may have difficulties fulfilling. As a recent survey presented by Becerril *et al*. [46] showed that more than two thirds of Diamond OA journals not indexed in DOAJ “comply with best practice guidelines”, it is likely many of these journals may in fact already be compliant to registration in DOAJ. Their absence from DOAJ may therefore be explained by a lack of awareness of its existence or importance among their editorial teams, or of time and resources for undertaking the application process. A recent study by Krapež [47] showed journals published by non-commercial publishers generally have less access to publication-related services.

### A different manifestation of the oligopoly

Although the position of the traditional major publishers, such as Elsevier, Springer Nature, Wiley and Taylor & Francis, may appear weakened in terms of sheer publishing numbers, they still represent a major share of the most visible research, managing to perpetuate the symbolic capital associated with their journals. In addition, the dominance of the major publishers may nowadays be oriented differently. For more than a decade, they have aimed to diversify their products, often based on artificial intelligence (AI) [7], and they will likely continue to do so. For example, Elsevier has a long record of developing applications: management and showcasing of research and publications (ScienceDirect; Digital Commons), an information system for tracking faculty accomplishments (Pure), a bibliometric database (Scopus), reference software (Mendeley), a monitor for research performance (SciVal), a preprint repository (SSRN) and scientific document text mining applications (Fingerprint Engine), among others [48]. Commercial publishers have focused on AI applications for some period, with a growing interest in building tools powered by Large Language Models trained on, at least partly, their own corpus.

## Conclusions

Globally, the major scientific publishers may have lost their dominance in terms of journal and article volumes, likely since the early 2000s, and mainly due to the explosive growth in journals and articles published elsewhere. However, they remain in control of the journals that are considered as the most prestigious, as shown by their dominant share of papers indexed in WoS. Mirroring the development of the Web from the 1990s onward, the emergence of online publishing tools such as OJS has likely been one of the main factors contributing to the diversification of journal ownership. The potential of literature findability and accessibility has increased markedly, which may have been to the advantage of independent journals. Moreover, these are more frequently available in OA, particularly beneficial to a readership from countries with restricted access to paywalled content, as well as to practitioners and decision-makers. In addition, the creation and the development of inclusive databases, such as Dimensions, which offers a different picture of the academic publishing landscape, has enabled us to identify and study the emergence of independent publishers. Providing such visibility to recently created, independent journals may favour their growth and enhance the global scholarly bibliodiversity. This diversity will benefit to communities relying particularly on content from independent publishers, such as countries in the Global South and practitioners in general, especially those using languages other than English, aided by a relatively high proportion of OA content. However, the growth of independent journals remains hindered by the research community’s capacity to move beyond journal’s imprint in its research evaluation practices.

## Supporting information

S1 Table Standardized publisher names and variants. Standardized publisher names and one or multiple variants of these, along width the number of articles retrieved using Dimensions and WoS, as well as the type of organization, either “Publisher”, “Aggregator” or “Questionable”.
(XLSX)
